# Supplementary material for: Daphnetin, a Coumarin with Anticancer Potential against Human Melanoma: In Vitro Study of Its Effective Combination with Selected Cytostatic Drugs
Source: Cells. 2023 Jun 9;12(12):1593. doi: 10.3390/cells12121593 (PMC10297575; doi:10.3390/cells12121593)
Supplement: Supplementary file 1 [file cells-12-01593-s001.zip › cells-2357748-supplementary.pdf]

Supplementary Materials

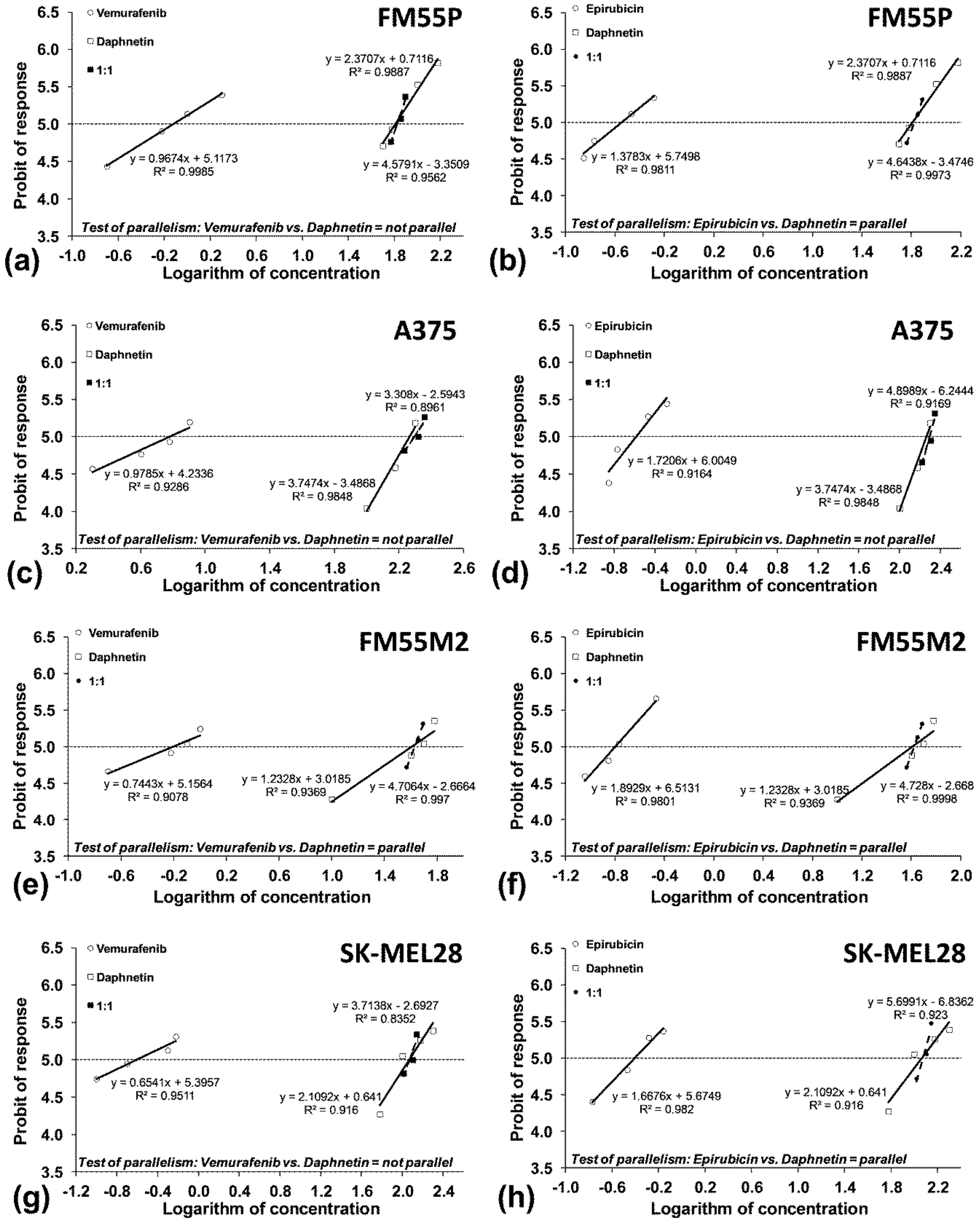

Figure S1. Concentration–effect lines (due to the computer-assisted log-probit method) for daphnetin (DAP) and vemurafenib (VEM) (a,c,e,g); daphnetin (DAP) and epirubicin (EPR) (b,d,f,h) administered alone and in combinations in the fixed-ratio of 1:1, illustrating the anti-proliferative effects of the drugs in the malignant melanoma cell lines: FM55P (a,b), A375 (c,d) FM55M2 (e,f) and SK-MEL28 (g,h) measured in vitro by the MTT assay.

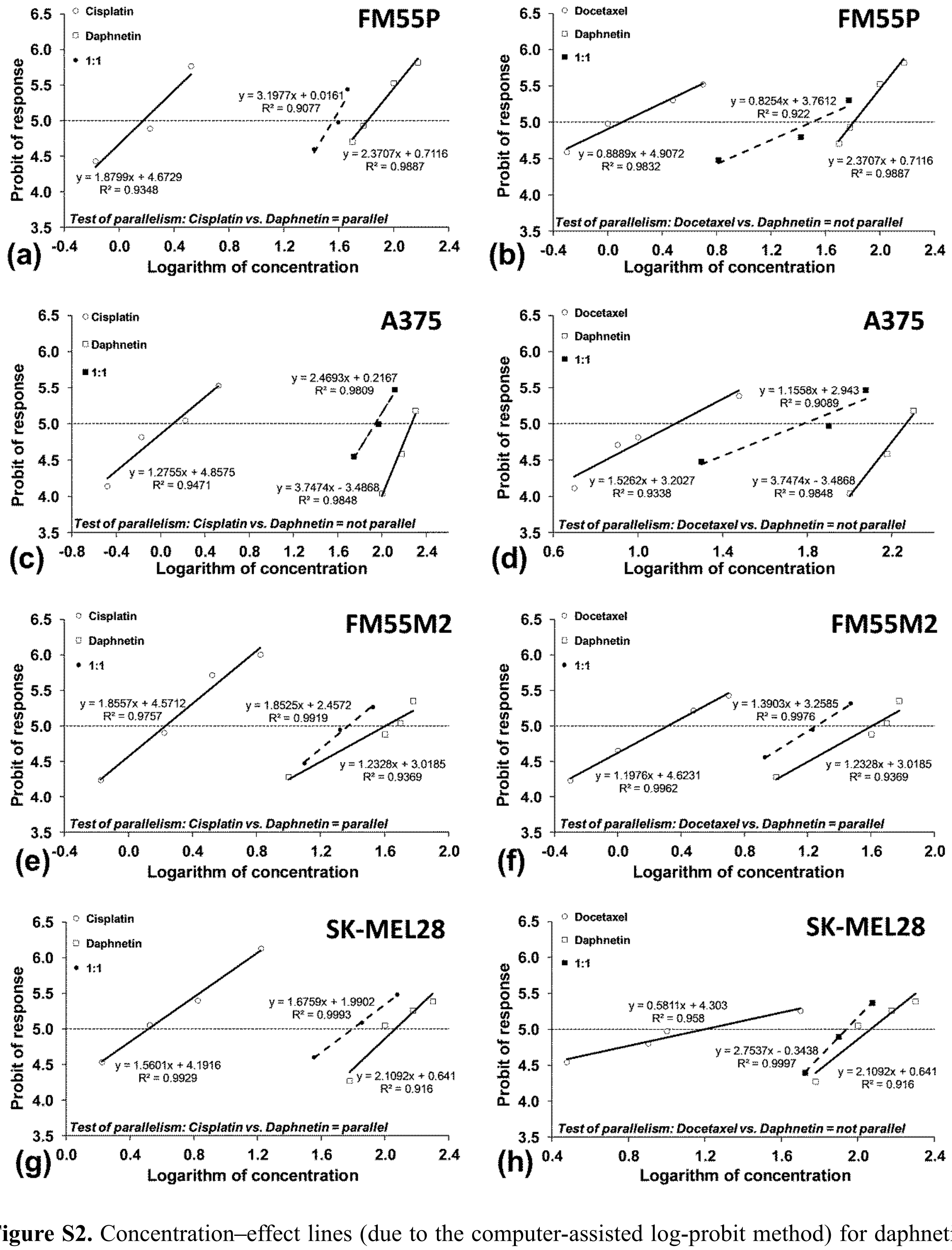

Figure S2. Concentration–effect lines (due to the computer-assisted log-probit method) for daphnetin (DAP) and cisplatin (CDDP) (a,c,e,g); daphnetin (DAP) and docetaxel (DOCX) (b,d,f,h) administered alone and in combination in the fixed-ratio of 1:1, illustrating the anti-proliferative effects of the drugs in the malignant melanoma cell lines: FM55P (a,b), A375 (c,d) FM55M2 (e,f) and SK-MEL28 (g,h) measured in vitro by the MTT assay.

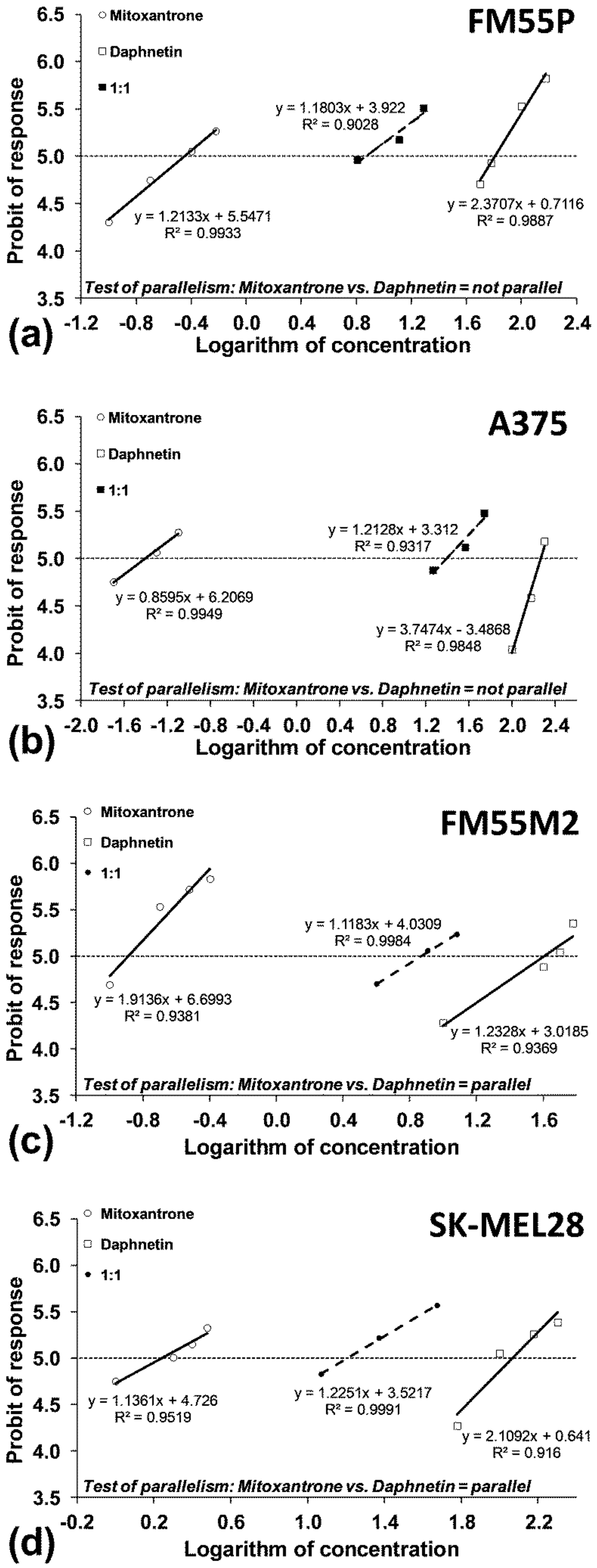

FM55P
Mitoxantrone
Daphnetin
1:1
y = 1.1803x + 3.922
R² = 0.9028
y = 1.2133x + 5.5471
R² = 0.9933
y = 2.3707x + 0.7116
R² = 0.9887
Test of parallelism: Mitoxantrone vs. Daphnetin = not parallel
Probit of response
Logarithm of concentration
(a)
A375
Mitoxantrone
Daphnetin
1:1
y = 1.2128x + 3.312
R² = 0.9317
y = 0.8595x + 6.2069
R² = 0.9949
y = 3.7474x - 3.4868
R² = 0.9848
Test of parallelism: Mitoxantrone vs. Daphnetin = not parallel
Probit of response
Logarithm of concentration
(b)
FM55M2
Mitoxantrone
Daphnetin
1:1
y = 1.1183x + 4.0309
R² = 0.9984
y = 1.9136x + 6.6993
R² = 0.9381
y = 1.2328x + 3.0185
R² = 0.9369
Test of parallelism: Mitoxantrone vs. Daphnetin = parallel
Probit of response
Logarithm of concentration
(c)
SK-MEL28
Mitoxantrone
Daphnetin
1:1
y = 1.1361x + 4.726
R² = 0.9519
y = 1.2251x + 3.5217
R² = 0.9991
y = 2.1092x + 0.641
R² = 0.916
Test of parallelism: Mitoxantrone vs. Daphnetin = parallel
Probit of response
Logarithm of concentration
(d)

Figure S3. Concentration–effect lines (due to the computer-assisted log-probit method) for daphnetin (DAP) and mitoxantrone (MTX) (a,b,c,d), administered alone and in combination in the fixed-ratio of 1:1, illustrating the anti-proliferative effects of the drugs in the malignant melanoma cell lines: FM55P (a), A375 (b) FM55M2 (c) and SK-MEL28 (d) measured in vitro by the MTT assay.

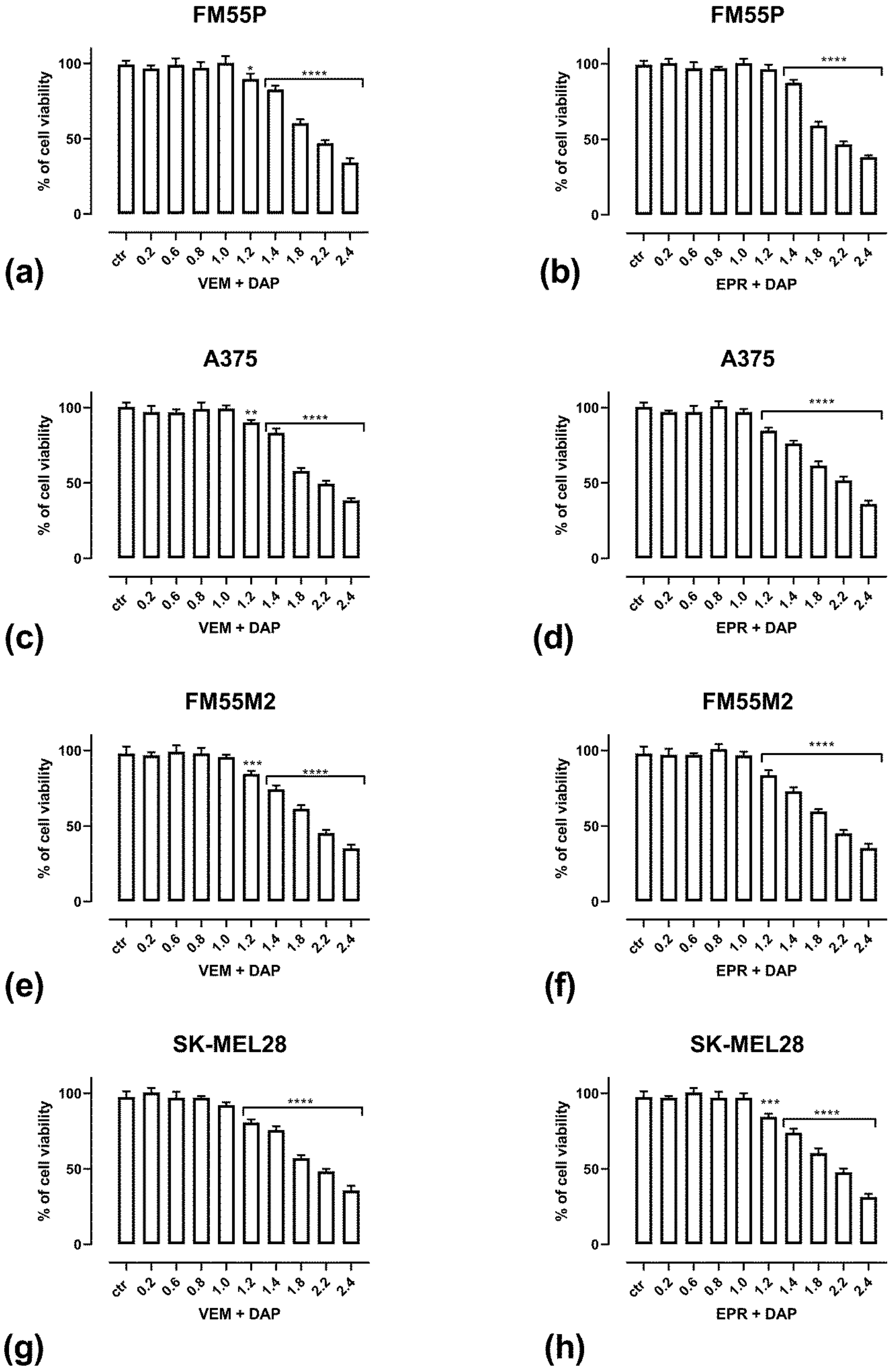
FM55P
% of cell viability
0
50
100
ctr 0.2 0.6 0.8 1.0 1.2 1.4 1.8 2.2 2.4
VEM + DAP
*
****
(a)
FM55P
% of cell viability
EPR + DAP
****
(b)
A375
% of cell viability
VEM + DAP
**
****
(c)
A375
% of cell viability
EPR + DAP
****
(d)
FM55M2
% of cell viability
VEM + DAP
***
****
(e)
FM55M2
% of cell viability
EPR + DAP
****
(f)
SK-MEL28
% of cell viability
VEM + DAP
****
(g)
SK-MEL28
% of cell viability
EPR + DAP
***
****
(h)

Figure S4: The anti-proliferative effect of VEM+DAP and EPR+DAP administered in combination against melanoma cell lines. Inhibition of cell proliferation was measured by the MTT assay after 72 h treatment with various concentrations of active agents. The anti-proliferative effect of VEM and DAP (a,c,e,g) and EPR and DAP (b,d,f,h) administered in combinations (at the fixed-ratio of 1:1 for the two-drug mixture) in increasing concentrations. All melanoma cell lines were exposed to VEM+DAP and EPR+DAP mixture treatment using different ratios of IC_{50} values (i.e., 1.0 indicates a half of the IC_{50} of VEM + a half of the IC_{50} of DAP; or a half of the IC_{50} of EPR + a half of the IC_{50} of DAP, respectively). The columns represent the mean for each concentration of mixture ± SEM. (**** $p < 0.0001$, *** $p < 0.001$, ** $p < 0.01$ and * $p < 0.05$ vs. the control (ctr) group).

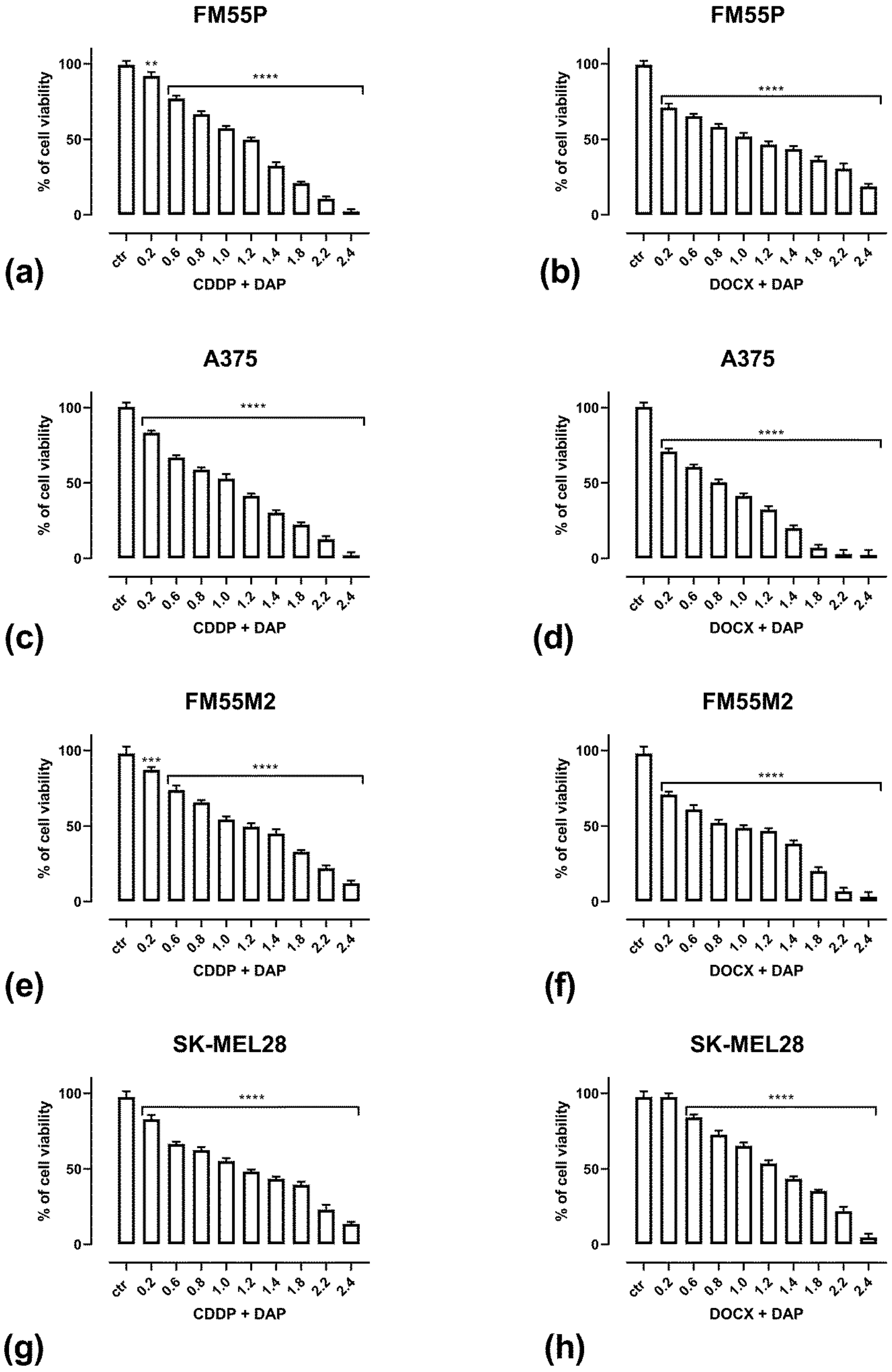

FM55P
% of cell viability
100
50
0
**
****
ctr
0.2
0.6
0.8
1.0
1.2
1.4
1.8
2.2
2.4
CDDP + DAP
(a)
FM55P
% of cell viability
****
DOCX + DAP
(b)
A375
% of cell viability
****
CDDP + DAP
(c)
A375
% of cell viability
****
DOCX + DAP
(d)
FM55M2
% of cell viability
***
****
CDDP + DAP
(e)
FM55M2
% of cell viability
****
DOCX + DAP
(f)
SK-MEL28
% of cell viability
****
CDDP + DAP
(g)
SK-MEL28
% of cell viability
****
DOCX + DAP
(h)

Figure S5. The anti-proliferative effect of CDDP+DAP and DOCX+DAP administered in combination against melanoma cell lines. Inhibition of cell proliferation was measured by the MTT assay after 72 h treatment with various concentrations of active agents. The anti-proliferative effect of CDDP and DAP (a,c,e,g) and DOCX and DAP (b,d,f,h) administered in combinations (at the fixed-ratio of 1:1 for the two-drug mixture) in increasing concentrations. All melanoma cell lines were exposed to CDDP+DAP and DOCX+DAP mixture treatment using different ratios of IC_{50} values (i.e., 1.0 indicates a half of the IC_{50} of CDDP + a half of the IC_{50} of DAP; or a half of the IC_{50} of DOCX + a half of the IC_{50} of DAP, respectively). The columns represent the mean for each concentration of mixture ± SEM. (**** $p < 0.0001$, *** $p < 0.001$, ** $p < 0.01$ and * $p < 0.05$ vs. the control (ctr) group).

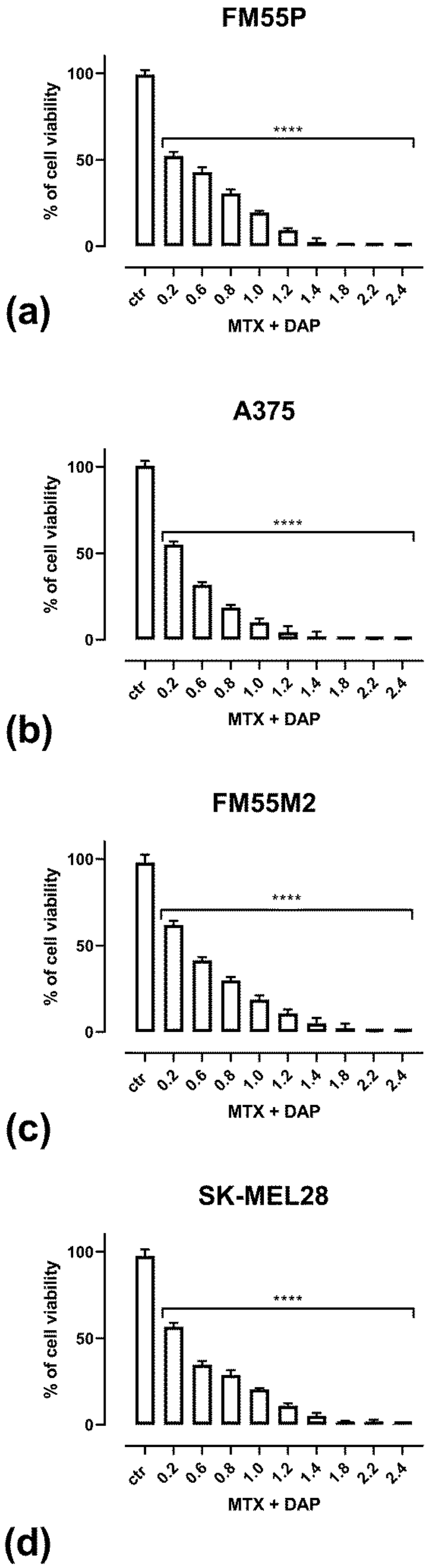
FM55P
% of cell viability
100
50
0
****
ctr
0.2
0.6
0.8
1.0
1.2
1.4
1.8
2.2
2.4
MTX + DAP
(a)
A375
% of cell viability
100
50
0
****
ctr
0.2
0.6
0.8
1.0
1.2
1.4
1.8
2.2
2.4
MTX + DAP
(b)
FM55M2
% of cell viability
100
50
0
****
ctr
0.2
0.6
0.8
1.0
1.2
1.4
1.8
2.2
2.4
MTX + DAP
(c)
SK-MEL28
% of cell viability
100
50
0
****
ctr
0.2
0.6
0.8
1.0
1.2
1.4
1.8
2.2
2.4
MTX + DAP
(d)

Figure S6. The anti-proliferative effect of MTX+DAP administered in combination against malignant melanoma cell lines. Inhibition of cell proliferation was measured by the MTT assay after 72 h treatment with various concentrations of active agents. The anti-proliferative effect of MTX and DAP (a,b,c,d) administered in combination (at the fixed-ratio of 1:1 for the two-drug mixture) in increasing concentrations. All melanoma cell lines were exposed to MTX+DAP mixture treatment using different ratios of IC_{50} values (i.e., 1.0 indicates a half of the IC_{50} of MTX + a half of the IC_{50} of DAP). The columns represent the mean for each concentration of mixture ± SEM. (**** $p < 0.0001$, *** $p < 0.001$, ** $p < 0.01$ and * $p < 0.05$ vs. the control (ctr) group).
